# Core Data Elements in Acute Myeloid Leukemia: A Unified Medical Language System–Based Semantic Analysis and Experts’ Review

**DOI:** 10.2196/13554

**Published:** 2019-08-12

**Authors:** Christian Holz, Torsten Kessler, Martin Dugas, Julian Varghese

**Affiliations:** 1 Institute of Medical Informatics University of Münster Münster Germany; 2 Department of Medicine A University Hospital of Münster Münster Germany

**Keywords:** common data elements, UMLS, acute myeloid leukemia, medical informatics

## Abstract

**Background:**

For cancer domains such as acute myeloid leukemia (AML), a large set of data elements is obtained from different institutions with heterogeneous data definitions within one patient course. The lack of clinical data harmonization impedes cross-institutional electronic data exchange and future meta-analyses.

**Objective:**

This study aimed to identify and harmonize a semantic core of common data elements (CDEs) in clinical routine and research documentation, based on a systematic metadata analysis of existing documentation models.

**Methods:**

Lists of relevant data items were collected and reviewed by hematologists from two university hospitals regarding routine documentation and several case report forms of clinical trials for AML. In addition, existing registries and international recommendations were included. Data items were coded to medical concepts via the Unified Medical Language System (UMLS) by a physician and reviewed by another physician. On the basis of the coded concepts, the data sources were analyzed for concept overlaps and identification of most frequent concepts. The most frequent concepts were then implemented as data elements in the standardized format of the Operational Data Model by the Clinical Data Interchange Standards Consortium.

**Results:**

A total of 3265 medical concepts were identified, of which 1414 were unique. Among the 1414 unique medical concepts, the 50 most frequent ones cover 26.98% of all concept occurrences within the collected AML documentation. The top 100 concepts represent 39.48% of all concepts’ occurrences. Implementation of CDEs is available on a European research infrastructure and can be downloaded in different formats for reuse in different electronic data capture systems.

**Conclusions:**

Information management is a complex process for research-intense disease entities as AML that is associated with a large set of lab-based diagnostics and different treatment options. Our systematic UMLS-based analysis revealed the existence of a core data set and an exemplary reusable implementation for harmonized data capture is available on an established metadata repository.

## Introduction

### Background

Medical documentation is complex and time-consuming. In routine documentation, it accounts for approximately 25% of a physician’s workload and demands as much time as direct patient care [1] and even more in study cases [2]. All patients with acute myeloid leukemia (AML) are to be treated within studies, following expert panel recommendations [3]. The number of patients with AML is relatively low with an incidence rate of around 3.7 per 100,000 in Europe [4]. The 5-year survival rate is below 50% [4]. Diagnostics and therapy comprise complex, repetitive laboratory analyses of different specimens at different points in time, chemotherapy cycles and schemes, donor search and selection, stem cell transplants, immunosuppressive therapy, repetitive follow-up examinations, and ongoing monitoring throughout the years of survival. All these are performed at different sites across Germany, Europe, and worldwide, depending on the hospitals’ facilities, donor selection, study group, and others. The complexity of the documentation process is obvious. In 2016, there were 4 AML study groups in Germany, that is, the *AML Kooperative Gruppe*, the *Deutsche Studieninitiative Leukämie*, the *AML Study Group*, and the *Ostdeutsche Studiengruppe für Hämatologie und Onkologie*. The European Leukemia Network (ELN) comprises more than 60 participating study centers. In 2016, there were 85 ongoing phase II or III trials for AML for adults listed in the European Union Clinical Trials Register for Germany (236 trials for the whole of Europe).

Clinical trial documentation itself is typically extensive and time-consuming [5]. In clinical trials, more than 1000 items such as laboratory values, vital signs, and diagnostic tests are collected per patient [6]. The number of pages in case report forms (CRFs) per trial has risen from 55 to 180 during the past years [5]. Study assistants are employed to reenter routine data into study CRFs manually, although automatic comparison and transformation is technically possible with minor limitations [7]. In our case, technical assistants fill out the transplant-specific forms of the German Zentrales Knochenmarkspender-Register für die Bundesrepublik Deutschland and the European Society for Blood and Marrow Transplantation (EBMT) with routine data by hand. Study data from CRFs of the Study Alliance Leukemia (SAL) are transferred into the SAL register manually. This approach is error prone. Owing to the relatively low incidence of AML, there is no quality management or certification process as it is common in other entities such as breast, prostate, colon, or other cancers.

Nowadays, special documentation assistants are employed to transfer routine data into software tools such as *ONDIS*, which is used in the administrative district of the Kassenärztliche Vereinigung Westfalen-Lippe. Both university clinics participating in this work are situated within this district. ONDIS serves as a tool for complete case documentation and quality management for manifestations of primary solid tumors but is also used for AML as, to our knowledge, there is no other option available on the market that provides the export and transfer of data to the epidemiologic cancer registries.

In 2013, Ries et al [8] stated that none of the existing German cancer datasets meet clinical documentation reality, even though they were already used as a base for cancer documentation, which is required by German law. To our knowledge, there are 2 datasets implemented in Germany, one by the Gesellschaft der Epidemiologischen Krebsregister in Deutschland e.V. and the other one by the Arbeitsgemeinschaft Deutscher Tumorzentren (ADT). They were established in 2008, revised in 2014, and are under ongoing modifications. Today, there are special datasets for breast, prostate, colon, glioma, and some other cancers, but there is none for leukemia. The 2018 ADT core dataset itself does not reflect on cancers without the manifestations of primary solid tumors, such as AML. Thus, it seems that no core dataset for AML documentation exists so far.

The layout and content of forms, regardless of which documentation context, organization, or medium, are mostly kept as intellectual property of the particular organization. This applies to standard forms of routine documentation in hospitals, CRFs in clinical or epidemiological studies performed by study groups, and register forms of national and international registries. They are not accessible to the public [9]. In addition, the mode of documentation is varying. Patient care forms often comprise free-text elements, whereas clinical trial documentation is structured on a higher level [2]. The reuse potential of information is generally higher if the original data are documented in a structured way [10,11].

The redundancy level of documentation within different documentation contexts is high [5]. Even the German Ministry of Health already recognized that large amounts of data are gathered redundantly and that cost-benefit analyses are recommendable [12]. It was proofed that digitalization of paper-based forms may not only reduce the workload for physicians in their daily routine by reducing redundant documentation [13] but may also generally improve the approach to structured documentation, facilitating improved accessibility, interoperability, and analysis of data [14]. Ongoing studies on interoperability standards of different documentation solutions are important and valuable for standardization of structured documentation [13] and secondary use of data, for example, in the scope of studies [15-17]. Structured documentation through the use of common data elements (CDEs) can improve data quality and data sharing [18]. The collection of detailed information of every single AML case is essential for patient surveillance [19]. Previous work already showed the benefit that can be achieved if all patients’ documentation is semantically annotated in cancers of the breast and prostate [2].

### Objectives

The aim of this work was to search for CDEs of AML documentation in clinical routine, registries, and studies. It focuses on the methods to create and provide standards for documentation and CDEs. It extends the previous collection of key data elements for myeloid leukemia, which has undergone clinical evaluation by several hematologists [13] and now focuses on specific data items for AML based on a larger dataset.

A medical concept is a semantic identifier to encode the medical information that is required by the documentation of an item. The item *patient performance status*, for example, is encoded by the concept *ECOG performance status, UMLS C1520224*. By adding the type of data and possible values to the concepts, a list of CDEs is created [20]. This list is usable to harmonize documentation of different contexts and to facilitate improved interoperability between health information systems.

The systematic analysis is performed on a set of different forms collected by the authors and semantically enriched using Unified Medical Language System (UMLS) codes [21]. The collection contains sets of AML documentation from 2 German university hospitals, international clinical AML studies performed by 3 study groups, national and international register forms, and a de facto international standard published previously by the ELN [3].

On the basis of the comparison of documentation forms, the following questions are addressed:

1. What are the most frequently used medical concepts in AML documentation?
2. To which degree do the register, routine, and clinical trial documentation represent or meet the ELN standard?
3. To which extent do routine, clinical trial, and register documentation overlap?
4. Do the sets of routine documentation of different hospitals differ (Bochum and Münster)? To which extent do datasets of register match with each other (EBMT and SAL)?

## Methods

### Data Collection

Different documentation contexts of AML were identified based on previous reports to represent a wide range of routine and research documentation on AML [13], which are listed in Table 1.

The collection of forms was performed between December 2015 and October 2016. A total of 2 university hospitals provided their electronic routine documentation forms and we chose 11 discharge letters—reviewed by a hematologist and deemed representative and complete regarding documentation items—out of the collection of cases of the previous 24 months. They were anonymized before the analysis started. Overall, 15 routine documentation forms such as laboratory reports, medical history, diagnostic finding, and stem cell transplant forms of both hospitals were collected and manually compared against the discharge letters. In total, 8 of them were annotated. In addition, 2 study groups from Germany and the Netherlands provided complete CRFs of 7 national or international studies. Furthermore, 3 registries of different sizes were identified via an Web-based query and by contacting the hematologist-oncologists. Their forms were collected. All right holders agreed to the analysis of forms and parts of the forms were publicly available. All documents were checked for integrity by 2 hematologist-oncologists familiar with AML therapy, documentation, and studies. Table 1 shows the different documentation contexts the forms were assigned to and their numbers.

**Table 1:** Documentation context and forms in each field.

Documentation context	Number of sources
Routine documentation	11 comprehensive, representative discharge letters of 2 university hospitals (Routine BO^a^+Routine MS^b^); 15 forms of routine documentation of 2 university hospitals (8 semantically annotated)
Registries	2 (EBMT^c^, SAL^d^-AML^e^)
Studies	3 (all case report forms of HOVON 132^f^, AML-AZA^g^, AMLSG 21-13^h^)
Quality measurement	None (not existing)
Recommendations of official associations	1 (European Leukemia Network recommendations [3])

^a^Routine BO: University Hospital Bochum-Langendreer.

^b^Routine MS: University Hospital of Münster.

^c^EBMT: Register by the European Society for Blood and Marrow Transplantation.

^d^SAL: Study Alliance Leukemia.

^e^AML: acute myeloid leukemia.

^f^HOVON 132: Haemato Oncology Foundation for Adults in the Netherlands, Study 132.

^g^AML-AZA: a randomized, multi-center phase II trial to assess the efficacy of 5-azacytidine added to standard primary therapy in elderly patients with newly diagnosed AML of University Münster.

^h^AMLSG 21-13: Deutsch-Österreichische Studiengruppe Akute Myeloische Leukämie, Study 21-13.

**Figure 1:**
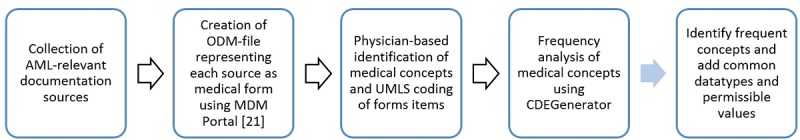
Process of creating common data elements. AML: acute myeloid leukemia; ODM: Operational Data Model; MDM: Medical Data Models; UMLS: Unified Medical Language System.

### Data Analysis

#### Semantic Form Annotation

The overall process is illustrated in Figure 1. All collected documentation models (see Table 1) were mapped into the Operational Data Model (ODM), defined by the Clinical Data Interchange Standards Consortium (CDISC). The Medical Data Models Portal (MDM-Portal) [22] served as a Web framework for creating ODM files using the ODM editor (University of Münster) [6] to standardize the input forms and to manually add semantic codes for form items. Semantic codes were chosen from the UMLS meta-thesaurus by a medical expert, based on the existing coding principles [23]. Medical concepts were manually extracted from the discharge letters, which are naturally free-text letters, and then semantically annotated with UMLS codes.As the coding principles indicate, pre and postcoordinated codes were chosen per item. If no precoordinated code was available for a medical concept, postcoordination was considered. Items with nonmedically relevant data (eg, *page number*) or insignificant content such as *other*, *specify*, or *further comment* were ignored.

#### Semiautomated Analysis

The manually UMLS-coded ODM forms were uploaded to the MDM-Portal and made publicly available. A second review that was followed by a UMLS-experienced physician ensured the quality of the coded concepts. Disagreements in coding were discussed between physicians regarding coding principles [23] and the frequency rate–assisted MDM-Portal ODM editor was used. The coded ODM forms were analyzed by CDEGenerator [13,24], an in-house implemented Java-based Web application. CDEGenerator automatically sorts medical concepts (eg, medication) of the existing data items according to their frequency (by counting identical UMLS codes) and also shows similarity of medical concepts based on the code overlaps of postcoordinated concepts, for example, *medication start date* is similar to *medication end date*, as the main concept *medication* is the same. An initial list of most frequent medical concepts and concept overlaps between all different forms was generated.

#### Generation of Common Data Elements

A list of most frequent medical concepts was generated by CDEGenerator by analyzing all ODM files and counting same UMLS codes. Concepts that were semantically similar (eg, birth date/age, gender/sex, and previous malignancy/tumor history) were grouped as one based on the expert’s decision.By adding to each medical concept its datatype and possible values, for example, codelist items, a medical concept also represents a data element [20]. Data elements that were documented coherently (eg, systolic and diastolic blood pressure) were grouped into item groups. A data element will be added to the resulting set of CDEs if it occurs at least twice within all sources or if it is listed in the standard published by the ELN [3]. The list was then checked by a medical expert to avoid any redundancies or important missing medical concepts. All CDEs and item groups were then mapped to documentation categories and implemented as standardized CDISC-ODM files and uploaded to the MDM-Portal for scientific discussions and reuse.

#### Pairwise Comparison of Documentation Contexts

The pairwise comparison of different documentation contexts can be made on different bases: (1) the comparison of different contexts such as routine and clinical trial documentation with each other; (2) the comparison of different sources of the same context, such as routine documentation of different origins/hospitals; (3) the overlap between the ELN standard and a combination of other contexts such as routine and clinical trial merged together.

CDEGenerator was used to identify common concepts of different sources or contexts and to output percentages of overlapping concepts.

## Results

### Overview

To identify a semantic core of frequently used medical concepts in routine and research documentation of AML, a total of 3265 medical concept occurrences were identified of which 3245 could be UMLS-coded (99.38%). After review of a second UMLS-experienced physician, 27 concepts (0.83%) were given different UMLS codes upon consensus decision. Among all concept occurrences, 1414 were unique medical concepts. The next section provides details on the frequency of concept occurrences.

### Cumulative Frequencies

**Figure 2:**
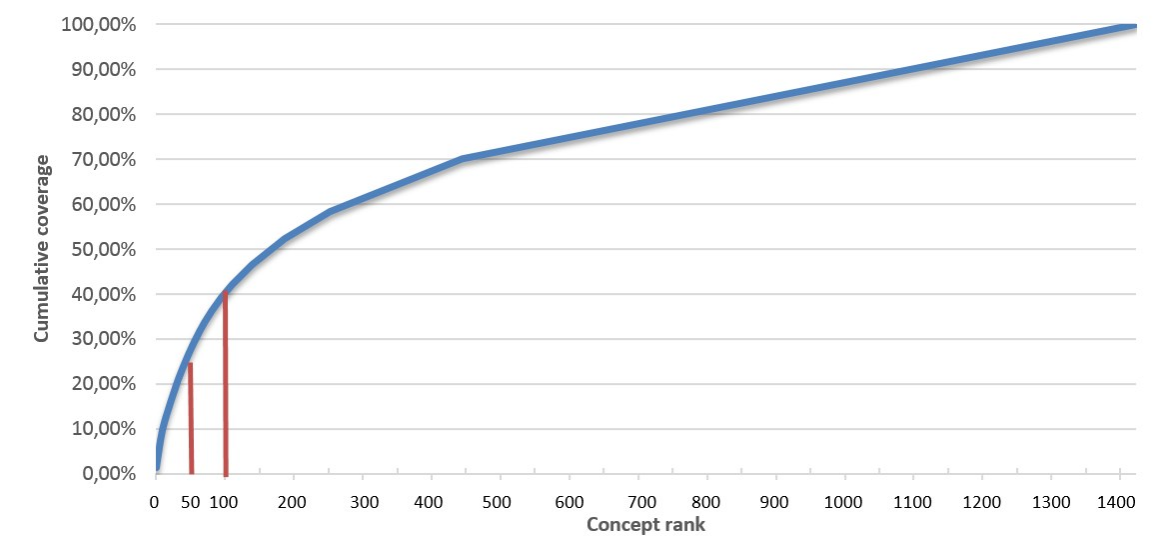
Cumulative frequency coverage of all different concepts. The 50 most common concepts cover about 27% of all concept occurrences, and the 100 most frequent concepts cover about 39.5% of all concept occurrences.

Among 1414 unique medical concepts, the 50 most frequent medical concepts cover 26.98% of all concept occurrences within the collected AML documentation. The top 100 concepts represent 39.48% of all concept occurrences. Figure 2 shows the cumulative frequencies.

### Unified Medical Language System Terminology and Acute Myeloid Leukemia

For about 1% (m=20) of the relevant medical concepts, no adequate UMLS code could be assigned, such as for the following codelist items: *matched related donor*, *matched unrelated donor*, *mismatched unrelated donor*, *HLA identical sibling*, *HLA identical parent*, and *2 or more antigen mismatched related donor* (all belonging to bone marrow transplantation donors). Concerning graft-versus-host disease status, items such as *resolved to baseline*, *resolved with sequelae*, *ongoing with higher CTCAE grade* were missing. Owing to the complexity of these concepts, postcoordination for these concepts was not applied to avoid information loss. In addition, certain AML-specific vocabulary is also missing—or may be underrepresented—in the UMLS terminology. The *WHO tumor classification*, for example, has a UMLS code but not the *WHO AML classification*. The following concepts were also missing in the UMLS databases at the time of the research: *EBMT risk score*, *clusters of blasts*, *−* *7q/7q mutation*, and *Hematopoietic Cell Transplantation-Comorbidity Index (HCT-CI)*. Some medical concepts have 2 different codes, such as *C1516728* *—* *Common Terminology Criteria for Adverse Events* and *C3888020* *—* *Common Terminology Criteria for Adverse Events*, even though the same concepts are meant.

### Generation of Common Data Elements

The generation of CDEs was realized by counting absolute frequencies of UMLS codes over all collected and annotated forms. Items represented in at least 2 different sources were added to the list of CDEs. UMLS codes found only in 1 single documentation source were excluded, even if used repeatedly there. Figure 2 provides an overview of documentation categories. All CDEs were implemented as CDISC-ODM files and are available with open-access on the MDM-Portal. The portal provides a number of conversions such as to REDCap (Research Electronic Data Capture) models and HL7 FHIR (Health Level Seven Fast Healthcare Interoperability Resource) questionnaires [25].

**Figure 3:**
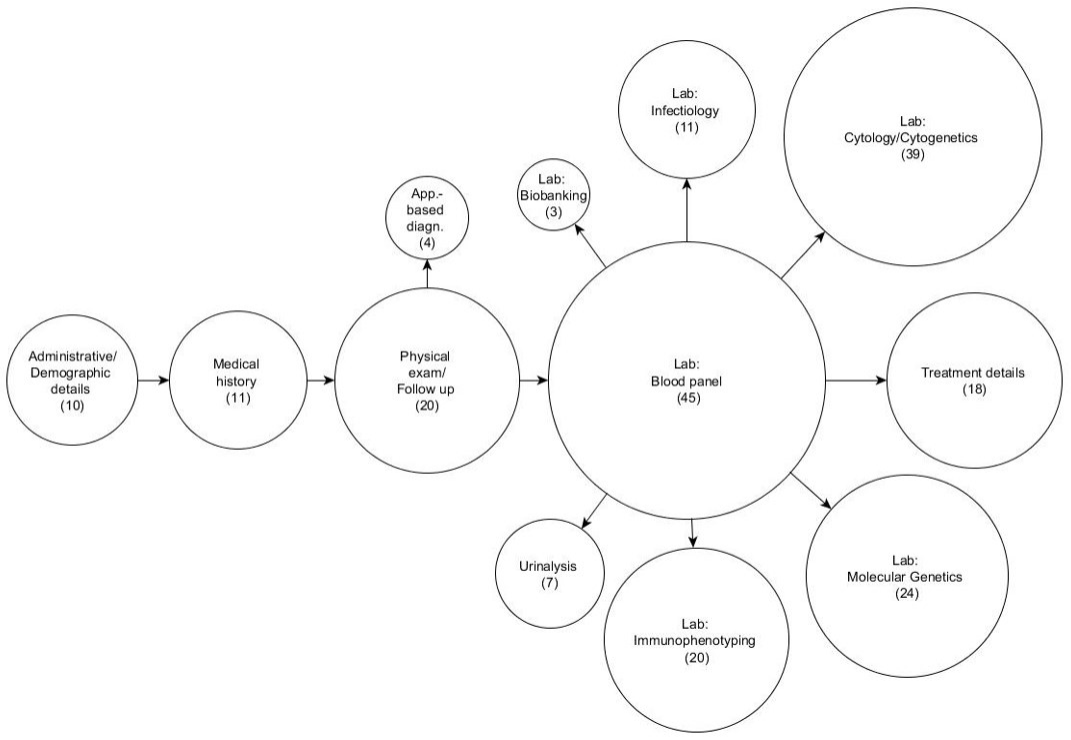
Documentation landscape of the common data elements (CDEs) of acute myeloid leukemia patients. Each circle represents a documentation category of the CDEs. The area of a circle corresponds to the number of data elements in that category. For example, there are 45 data elements within the laboratory blood panel, which represents the largest documentation category. A total of 212 CDEs were identified. App.-based diagn.: Apparatus-based diagnostics (eg, ultrasound and electrocardiogram).

We could show that the CDEs appeared in all medical categories throughout the patient therapy course. CDEs exist from the beginning to end of therapy (Figure 3).

The most frequently used concept of all documentation contexts is *disease response*. Table 2 shows a list of the 20 most CDEs relevant for AML therapy, their subconcepts, absolute concept frequency, and documentation context in which the concepts are represented in.

The top 30 laboratory concepts are presented separately in Table 3, analogous to Table 2. Unspecific data elements have been manually filtered, for example, *patient birth date*, *gender*, and *patient name*. A complete list of all concepts is found in Multimedia Appendix 1. Implementation of data elements according to Clinical Data Interchange Standards Consortium–Operational Data Model format is available in [25].

**Table 2:** Top 20 of the most frequent concepts sorted by absolute concept frequency.

Concept and subconcepts	Documentation category	ACF^a^	Documentation context			
			Routine	Register	Study	ELN^b^ standard
Disease response/remission status: Complete remission; Complete remission with incomplete hematologic recovery; Partial response; Complete remission cytogenetic; Complete remission molecular; Resistant disease; Partial remission recurrence/relapse; and death in aplasia	Treatment details	42	✓	✓	✓	✓
Treatment status: number of therapies since the last visit; treatment outside of a study, palliative+after end of treatment; did patient start protocol treatment; cycle treatment/action taken; current therapy; additional therapies since last follow-up; treatment given since last report; disease treatment (apart from donor cell infusion or other type of cell therapy); treatment for disease; and planned (planned before HSCT^c^ took place)+current therapy	Treatment details	24	✓	✓	✓	—^d^
Adverse event: adverse event; adverse event number; adverse event indicator; and description of adverse event	Treatment details	16	✓	—	✓	—
Platelet engraftment: date of engraftment; platelets self-sustaining; and platelets >x mg/dL	Treatment details	12	✓	—	✓	—
Neutrophil engraftment: date of engraftment; neutrophil self-sustaining; and neutrophils >x mg/dL at day	Treatment details	11	✓	—	✓	—
Chemotherapy cycle	Treatment details	12	—	✓	✓	—
Concomitant medication	Treatment details	11	—	—	—	—
Diagnosis: WHO^e^ classification; FAB^f^ classification; date of diagnosis; and first diagnosis	Physical examination/follow up	27	✓	✓	✓	✓
Patient performance status: Karnofsky index and ECOG^g^ performance status	Physical examination/follow up	19	✓	✓	✓	✓
Concomitant disease/comorbidity: comorbidity; baseline concomitant diseases; and concurrent severe and/or uncontrolled condition	Physical examination/follow up	17	✓	✓	✓	✓
Second malignancy/other tumor: previous tumor disease in history; preexisting solid tumor (chemotherapy required); secondary malignancy; and second primary malignancy	Physical examination/follow up	16	✓	✓	✓	—
Cause of death	Physical examination/follow up	10	✓	✓	✓	—
Diagnosis date	Physical examination/follow up	13	—	✓	✓	—
Survival status: alive; dead; and unknown (lost to follow-up)	Physical examination/follow up	12	—	✓	✓	—
Extramedullary manifestation of disease	Physical examination/follow up	15	✓	✓	✓	✓
Pregnancy	Physical examination/follow up	12	✓	✓	✓	—
Drug toxicity	Physical examination/follow up	12	✓	✓	✓	—
HSCT details: HSCT-indicator; HSCT-type; date of transplantation; relation to donor; and chimerism	Bone marrow transplant	16	✓	✓	✓	—
Previous chemotherapy/radiotherapy, antineoplastic protocols: year of chemotherapy/radiotherapy; chemotherapy medication; and radiotherapy specification	Medical history	11	—	✓	—	—
Concomitant medication	Treatment details	11	—	—	✓	—

^a^ACF: absolute concept frequency; n=1057.

^b^ELN: European Leukemia Network.

^c^HSCT: human stem cell transplant.

^d^Data element is not represented in the documentation context.

^e^WHO: World Health Organization.

^f^FAB: French-American-British-Classification.

^g^ECOG: Eastern Co-operative Oncology Group.

**Table 3:** Top 30 of the most frequent laboratory concepts sorted by absolute concept frequency.

Concept and subconcepts	Documentation category	ACF^a^	Documentation context			
			Routine	Register	Study	ELN^b^ standard
Platelets blood level	Laboratory: blood panel	13	✓	✓	✓	✓
Bilirubin blood level	Laboratory: blood panel	13	✓	✓	✓	✓
Platelets blood level	Laboratory: blood panel	13	✓	✓	✓	✓
White blood count / leukocytes	Laboratory: blood panel	12	✓	✓	✓	✓
GPT^c^	Laboratory: blood panel	11	✓	✓	✓	✓
Blood group	Laboratory: blood panel	11	✓	✓	✓	—^d^
Serum creatinine	Laboratory: blood panel	10	✓	✓	✓	✓
Lactat dehydrogenase	Laboratory: blood panel	9	✓	✓	✓	✓
INR^e^/Quick	Laboratory: blood panel	9	✓	—	✓	✓
Hemoglobin	Laboratory: blood panel	9	✓	✓	✓	—
aPTT^f^	Laboratory: blood panel	7	✓	—	✓	✓
Alkaline phosphatase	Laboratory: blood panel	7	✓	—	✓	—
GOT^g^	Laboratory: blood panel	7	✓	—	✓	✓
Uric acid	Laboratory: blood panel	7	✓	—	✓	✓
Cytogenetic examinations	Laboratory: cytology/cytogenetics/cytochemistry	13	✓	✓	✓	✓
Blast cells/blast	Laboratory: cytology/cytogenetics/cytochemistry	15	✓	✓	✓	✓
Bone marrow examination^h^	Laboratory: cytology/cytogenetics/cytochemistry	13	✓	✓	✓	✓
Monocytes	Laboratory: cytology/cytogenetics/cytochemistry	11	✓	✓	✓	—
Lymphocytes	Laboratory: cytology/cytogenetics/cytochemistry	10	✓	✓	✓	—
CD34^i^ positivity	Laboratory: cytology/cytogenetics/cytochemistry	10	✓	✓	✓	✓
Auer rods	Laboratory: cytology/cytogenetics/cytochemistry	9	—	—	✓	✓
Clusters of blasts	Laboratory: cytology/cytogenetics/cytochemistry	9	—	✓	✓	—
Karyotype	Laboratory: cytology/cytogenetics/cytochemistry	8	✓	✓	✓	✓
Eosinophils	Laboratory: cytology/cytogenetics/cytochemistry	8	✓	—	✓	—
Basophils	Laboratory: cytology/cytogenetics/cytochemistry	7	✓	—	✓	—
Promyelocytes	Laboratory: cytology/cytogenetics/cytochemistry	7	✓	—	✓	—
Metamyelocytes	Laboratory: cytology/cytogenetics/cytochemistry	7	✓	—	✓	—
CMV^j^ positivity	Laboratory: infectiology	10	✓	✓	✓	✓
Ebbstein-Barr virus positivity	Laboratory: infectiology	8	✓	✓	—	—
Urine protein	Laboratory: urinalysis	7	✓	—	✓	✓

^a^ACF: absolute concept frequency.

^b^ELN: European Leukemia Network.

^c^GPT: glutamate pyruvate transaminase.

^d^Data element is not represented in the documentation context.

^e^INR: international normalized ratio.

^f^aPTT: activated partial thromboplastin time.

^g^GOT: glutamic oxaloacetic transaminase.

^h^Subconcepts: bone marrow puncture; bone marrow sample; bone marrow sampling date; and bone marrow examination possible.

^i^CD34: cluster of differentiation 34.

^j^CMV: Cytomegalie virus.

**Table 4:** Overlaps of pairwise documentation contexts (A,B).

A	|*A*|	*B*	|*B*|	|A ∩ B|	|A ∩ B| / |A|, %	|A ∩ B| / |B|, %
Clinical trial documentation	752	Routine documentation	250	116	15.43	46.40
Clinical trial documentation	752	Registries	428	117	15.56	27.34
Clinical trial documentation	752	ELN^a^ standard	154	70	9.31	45.45
ELN standard	154	Routine documentation	250	46	29.87	18.40
ELN standard	154	Registries	428	36	23.38	8.41
Registries	428	Routine documentation	250	83	19.39	33.20
Routine Bochum	112	Routine Münster	138	106	94.64	76.81

^a^ELN: European Leukemia Network.

### Overlap Analysis for Pairwise Comparison of Documentation Contexts

Table 4 shows the result of the overlap analysis. Routine documentation (250 unique concepts), clinical trial documentation (752 unique concepts), registries (428 unique concepts), and ELN standard (154 unique concepts) are compared and show an overlap of 9% to 46%.

### Comparison of Routine and Clinical Trial Documentation

The clinical trial documentation comprises 752 different medical concepts, whereas the routine documentation comprises 250 concepts. Furthermore, 46.4% of the items in the routine documentation are also found in clinical trial documentation. Naturally, items such as *study site identifier/hospital ID* UMLS code C2825164 are found in study and register documentation but not in routine documentation. More therapy-specific items, such as *adverse event* C0877248, are concepts that can only be found in clinical trial documentation. Meanwhile, the existence of an *extramedullary manifestation* C1868812 is naturally of substantial medical interest and can therefore be found in all documentation areas and exists in all of those. *EBV-positivity* C0014644, *toxoplasmose-positivity* C0040558, or *CRP* C0201657 were relevant in routine documentations of both university hospitals but in none of the included CRFs of clinical trials.

### Clinical Trial Documentation and Registries

The registries analyzed in this work used 428 different concepts. The overlap of clinical trials documentation (752) and registries is 15.5% relating to clinical trial documentation and 27.3% relating to registries. Nearly one-third of the registries’ data can be found in the clinical trial documentation. *Concomitant medication* C2347852 is relevant for all clinical trials but not mentioned in registries. Again, *EBV-positivity* C0014644 is found in all registries and in routine documentation but in none of the studies.

### Comparison of European Leukemia Network Standard With Registries

By comparing the registries (428) with the ELN standard (154), overlaps of 23.3% with regard to registries and 8.4% with regard to the ELN standard were found. This was the lowest overlap found for all analyses performed in this study. Administrative and organizational items are missing in the ELN standard. Examinations are often only mentioned in the standard, but their detailed medical concepts are not all listed, for example, *hemoglobin* C0019046 can be found in all documentation fields but not the ELN standard. This also applies to entries regarding the therapy. Registries are mainly focused on the long-term aspects of the disease such as etiology or outcome/follow-up and much less on specific therapy-relevant lab parameters. Concepts such as blood hemoglobin concentration are not mentioned in registries but are of high importance in diagnostics and therapy of the disease.

### Comparison of Routine Documentation of 2 Hospitals

Finally, the routine documentations of the University Hospital Bochum-Langendreer and the University Hospital of Münster were compared, and routine documentation consisted of 112 and 138 medical concepts, respectively. The overlap of both is 94.6% and 76.8%, respectively. This amounts for the highest overlap of all analyses of this study. Items such as C0019196 and C0019159, which represent hepatitis C/A positivity, were only a part in one of the 2 hospitals’ routine documentation. The same applies to *D-dimer* C2826333, *blood gas analysis* C0005800, or *chloride* C0008203.

### Comparison of Clinical Trials and the European Leukemia Network Standard

Nearly half of the medical concepts of the international standard are found in the documentation of clinical trials. The clinical trial documentation consists of more than 700 medical concepts, 4 times more than the European Leukemia Network standard of around 150 medical concepts.

### Comparison of the European Leukemia Network Standard and Routine Documentation

In the routine documentation, around one-third of the items of the ELN standard are represented. One-fifth of the routine documentation items are found in the ELN standard. For instance, *date of birth/age* C1704632 are mentioned in both routine documentation and the ELN standard. *Blood group* C0005810, *weight* C0005910, and *magnesium* C0364745 are mentioned in routine documentation but not in the ELN standard. *t(v;11)(v;q23) mutation* C1515810, *nonspecific esterase* C0054741, or *prior exposure to toxic agents* C0014412 are found in the standard but not in routine documentation.

## Discussion

### Principal Findings

Documentation of AML is complex and time-consuming. The neoplastic disease has complex therapy options, a sophisticated chemotherapy regimen, and often the need for preparation and performance of stem cell transplantation. In addition, there is a need for matching cancer documentation guidelines and recommendations by law in Germany. The fact that most patients are treated within studies leads to further documentation arms.Different health care institutions are involved in the documentation process.The detailed analysis performed in this study could clearly show that the content of AML documentation is often quite redundant. Clinical trial documentation and routine documentation overlap by 42.6%. By establishing interfaces between those documentation contexts, information once gathered could be automatically synced. This clearly reduces the documentation effort.Across all documentation contexts in AML, a basic dataset of 50 CDEs was found to amount for 43.7% of all different medical concepts used. This relatively small number of items could be used as a core dataset. Reusing this semantically annotated dataset would reduce redundancy and costs when it would be made available to all documentation fields for automatic export. In practice, a dynamic database continuously updated with the most recent values of the CDEs could become source for automatic extraction of elements for other documentation arms such as registries, clinical trial documentation, and others. As a small practical example, requesting therapeutic drug levels could work in just 1 click. Today, it is often necessary to fill out forms with *patient weight*, *age*, *gender*, and *kidney test values* manually. On a large scale, high percentages of clinical trial documentation could be filled out automatically. Imagine your mobile phone’s autocomplete/word completion functionality. It enhances you to fill out specific forms and websites faster and more convenient by anticipating possible values and giving you option to choose these. Analogue case-specific completion of data in Electronic health records is feasible on a base of CDEs. At the same time, standardization and quality assurance would become easier to perform because of the transparency in documentation.

We could show that the semantic annotation of nearly a whole complex medical entity is feasible, by reaching an annotation rate of more than 98%.Semantic annotations mark the distinct, clear meaning of medical documentation items. Therefore, they enhance the possibilities of data integration and exchange [14,18]. Applying statistical tools to an annotated dataset can help identify missing medical concepts or solitary ones. Solitary items might be outdated, too. As an example, in our work, the concept *EBV-positivity* was mentioned during routine documentation and in registries but is not/no more of interest in research (study documentation). Thinking one step further, semantic annotation could open the doors for reusing data, for example, for studies with other aims (secondary use). Not only for scientific questions, but also for the daily routine of physicians, a fully annotated documentation is of practical value. Automatic generation of standardized discharge letters using dynamically filled text blocks means time-savings and improves quality and safety through structured documentation [2]. Additional benefit of an annotated documentation is the good searchability, even across different languages.

We noticed that blank medical forms of all documentation contexts are difficult to find and gain access to. As a strength of this work, personal contact with the authors of clinical trials, routine documentation, and registries was established and written consent to the usage was obtained. A higher level of awareness of the value needs to be reached.

We experienced what is known from other research: there is apparently no knowledge of the value of the blank CRFs [9].

### Limitations and Strengths

In this work, the process of extraction and annotation of items from discharge letters was performed and supervised by physicians. This ensured a high level of semantic quality of the generated data. A human medical professional can extract medical concepts out of free-text elements, tables, graphics, and other sources. Medical concepts had to be recognized, extracted, and annotated. This approach requires a lot of effort in terms of time, personal resources, and, in the end, noticeable costs.

The aim of this work was to create a dataset of high quality out of routine data. As further data models with new biomarkers and other relevant concepts will arise in the future, an alternative step would be to combine our methodology with a preceding natural language processing (NLP) pipeline to automatically analyze a larger set of >1000 documentation sources.

Our method was to annotate medical concepts manually with a high grade of precision. The technical route to match only conceptually identical items and not similar ones could explain a lower percentage in this specific comparison of documentation contexts than expected.

Our extended AML dataset has a high level of congruence to a general leukemia dataset, which has been previously published and checked by independent international hematologists for integrity and consistency [13]. Previous work of Miotto and Wang [26] identified 115 common possible data items in clinical trial feasibility of all studies registered on Clinicaltrials.gov based on a computational approach. Although majority of those are found in our collection (87.8%), only 20.3% of it are a part of Miotto and Wang’s list. None of our AML-specific laboratory items were found there, which indicates the specific focus on AML in this work.

Implementation of the generated standard dataset can be used for different purposes: automatic generation of text modules in discharge letters, automated filling of cancer database forms, or any other. Comparison of the dataset with that of other entities to generate and complement a general basic clinical trial dataset could be another aim. NLP as a supplemental tool for annotating CRFs or other forms might speed up the manual annotation process [27]. The quality of the annotations if not revised manually is of course questionable.

Assigning UMLS codes to medical concepts is dependent on the personnel performing the coding (interrater agreement) and the existence of highly similar codes [27]. In our case, the example of annotating the procedure or the result/value was questioned. One of the coders chose C0005821 *blood platelets*, the other agreed on C0032181 *platelet count measurement*, which was taken in the end. Our dataset can serve as a base for future annotations of AML CRFs.

### Conclusions

The lack of standardization and semantical annotation of documentation for patients with AML is obvious. A high percentage of the documentation is performed as free text, which makes reusing information impossible without a lot of effort. As our research shows, there is a high overlap of data in clinical trial and routine documentation, as well as in clinical trial and register documentation. We identified a semantic core of data items which has been implemented in a highly structured format and can guide as a base for harmonized and efficient data collection and secondary use.

The benefits of datasets for CDEs in other entities, not only neoplastic diseases, are obvious, especially widespread diseases such as cardiovascular, stroke, neurological, and others with the need of complex and/or long-term therapy can be addressed.

## Appendix

Multimedia Appendix 1 List of all coded medical concepts.

## Abbreviations

ACF: absolute concept frequency
ADT: Arbeitsgemeinschaft Deutscher Tumorzentren
AML: acute myeloid leukemia
aPTT: activated partial thromboplastin time
CD34: cluster of differentiation 34
CDE: common data element
CDISC: Clinical Data Interchange Standards Consortium
CMV: Cytomegalie virus
CRF: case report form
EBMT: European Society for Blood and Marrow Transplantation
ECOG: Eastern Co-operative Oncology Group
ELN: European Leukemia Network
FAB: French-American-British-Classification
GOT: glutamic oxaloacetic transaminase
GPT: glutamate pyruvate transaminase
HSCT: human stem cell transplant
INR: international normalized ratio
MDM: Medical Data Models
NLP: natural language processing
ODM: Operational Data Model
SAL: Study Alliance Leukemia
UMLS: Unified Medical Language System
WHO: World Health Organization
